# Supplementary material for: Synergistic Use of All-Acceptor Strategies for the Preparation of an Organic Semiconductor and the Realization of High Electron Transport Properties in Organic Field-Effect Transistors
Source: Polymers (Basel). 2023 Aug 13;15(16):3392. doi: 10.3390/polym15163392 (PMC10458505; doi:10.3390/polym15163392)

Supplementary Information

Compound 2: To a solution of compound **1** (2.00 g, 6.67 mmol) in anhydrous dimethylformamide (DMF, 50 mL), 11-(3-bromopropyl)henicosane (6.93 g, 16.67 mmol, 2.50 eq) and potassium carbonate (K_2CO_3, 2.76 g, 20.00 mmol, 3.00 eq) were added. The mixture was stirred at 110 °C for 12 h under argon. The solution was extracted with dichloromethane (DCM) and then washed with water and brine, and dried over with Na_2SO_4. After removal of the solvent under reduced pressure, the residue was purified by silica gel chromatography with eluent (PE: DCM = 4: 1) to afford the product **2** as an orange-red solid (4.21 g, 64.9 %). δ ^{1}H NMR (400 MHz, Chloroform-d) δ 8.93 (d, J = 3.8 Hz, 2H), 7.63 (d, J = 4.9 Hz, 2H), 7.31 – 7.27 (t, J = 3.6 Hz, 2H), 4.04 (t, J = 7.9 Hz, 4H), 1.75 – 7.61 (m, 4H), 1.37– 1.22 (m, 78H), 0.88 (t, J = 6.7 Hz, 12H). ^{13}C NMR (75 MHz, Chloroform-d) δ 161.34, 140.01, 135.27, 130.59, 129.81, 128.62, 107.72, 42.57, 37.16, 33.55, 31.94, 30.51, 30.10, 29.72, 29.67, 29.37, 27.07, 26.70, 22.70, 14.13. HR-MALDI-TOF: $[M+H]^+$ calcd for $C_{62}H_{104}N_2O_2S_2^+$: 972.7539; Found: 972.7539.

Compound 3: Compound **2** (3.00 g, 3.08 mmol) and N-bromosuccinimide (NBS, 1.12 g, 6.32 mmol, 2.05 eq) were added to 40 mL of chloroform. The mixture was stirred under argon at 60 °C for 0.5 h. After removal of the solvent under reduced pressure, the residue was purified by silica gel chromatography (PE: DCM = 5: 1) to afford the product **3** as a purple-red solid. (3.14 g, 90.2 %). ^{1}H NMR (300 MHz, Chloroform-d) δ 8.69 (d, J = 4.2 Hz, 2H), 7.24 (d, J = 4.3 Hz, 2H), 3.95 (t, J = 7.8 Hz, 4H), 1.75 – 1.62 (m, 4H), 1.42 – 1.21 (m, 78H), 0.88 (t, J = 6.5 Hz, 12H). ^{13}C NMR (75 MHz, $CDCl_3$) δ 161.41, 139.41, 135.31, 131.43, 131.19, 118.95, 108.04, 46.36, 37.77, 31.93, 31.89, 31.19, 29.99, 29.65, 29.56, 29.50, 29.37, 29.29, 26.19, 22.70, 22.67, 14.12. HR-MALDI-TOF: $[M+H]^+$ calcd for $C_{54}H_{87}Br_2N_2O_2S_2^+$: 1128.5749; Found: 1128.5742.

Table SI. Elemental analysis for $C_{68}H_{106}N_4O_2S_4$.

	**C (%)**	**H (%)**	**N (%)**	**S (%)**
Calcd	71.65	9.37	4.92	11.25
Found 1	71.03	9.29	4.65	12.10
Found 2	71.12	9.28	4.63	12.04
Average	71.08	9.28	4.64	12.07

Figure S1. GPC data (molecular weight distribution and cumulative percent curves) for PDPPTT-2Tz polymer.

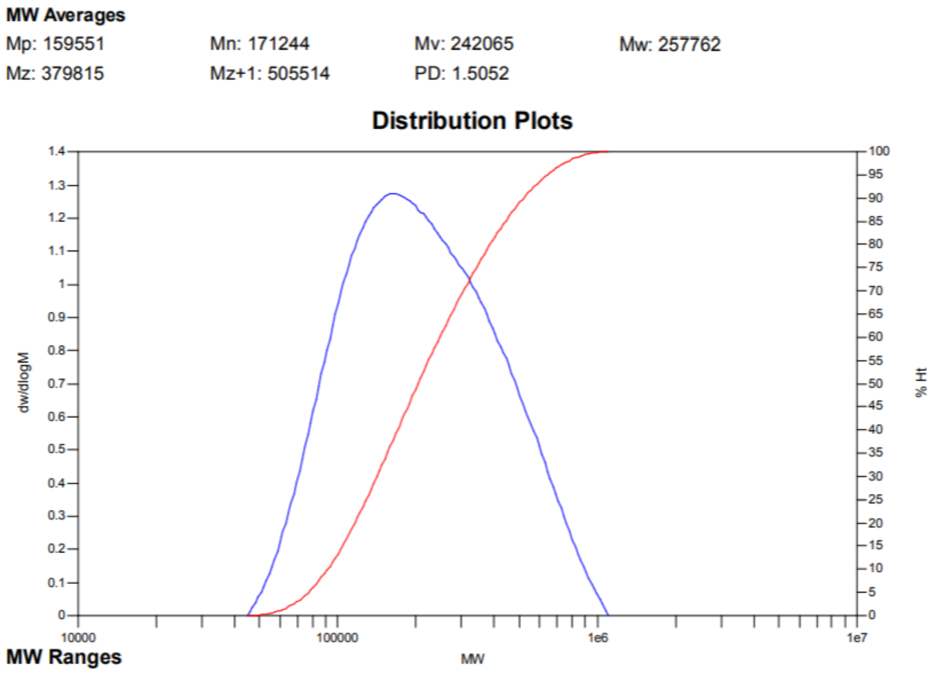

Figure S2. DSC of the PDPPTT-2Tz polymer in nitrogen.

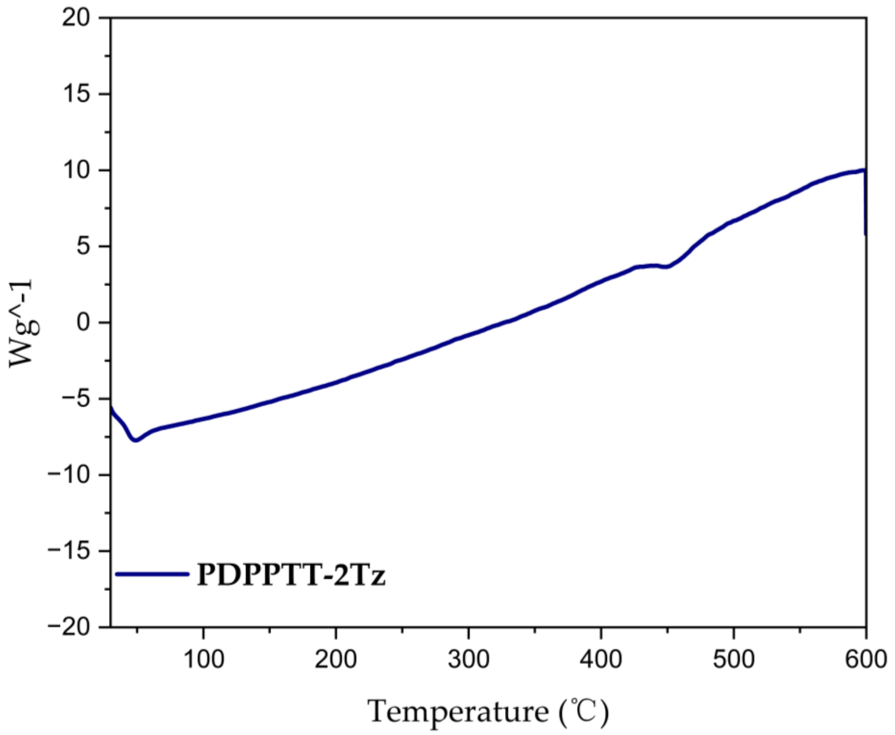

Figure S3. Infrared spectra of the PDPPTT-2Tz polymer.

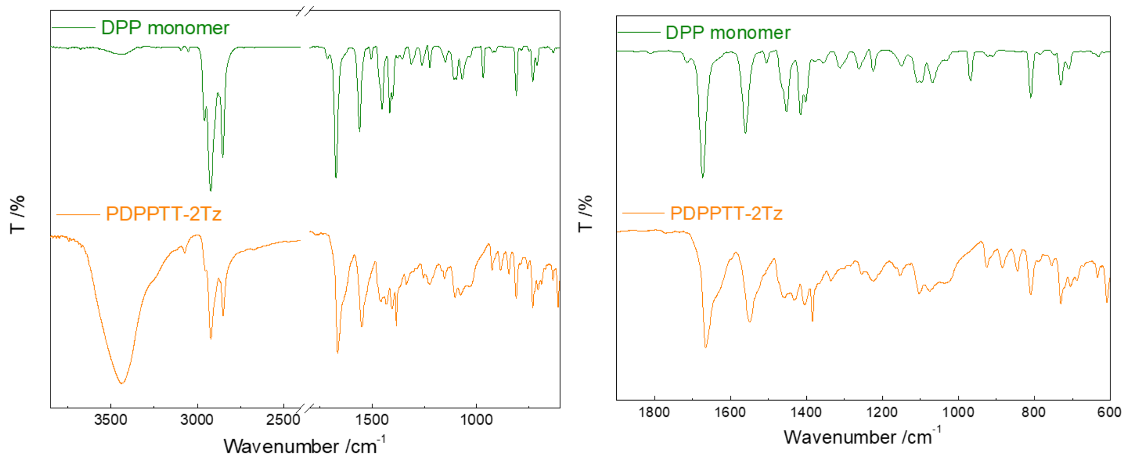

Figure S4. Electrostatic potential surfaces map.

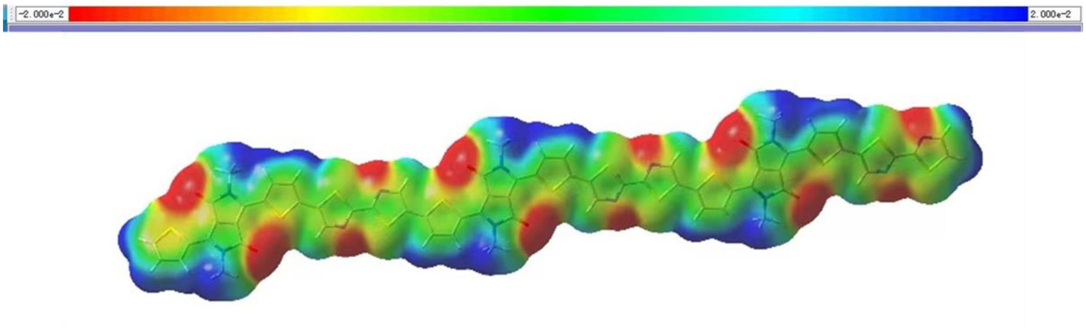

Figure S5. Molecular orbital maps.

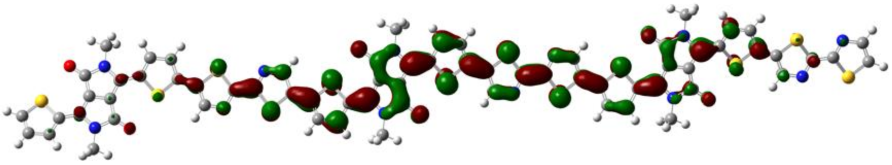

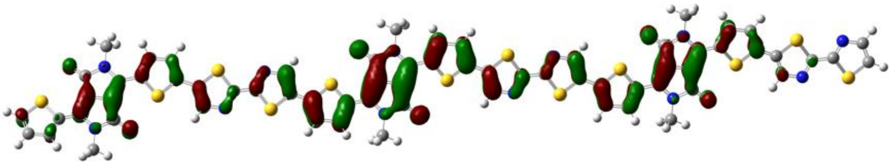

Figure S6. ^{1}H NMR spectra of Compound 2.

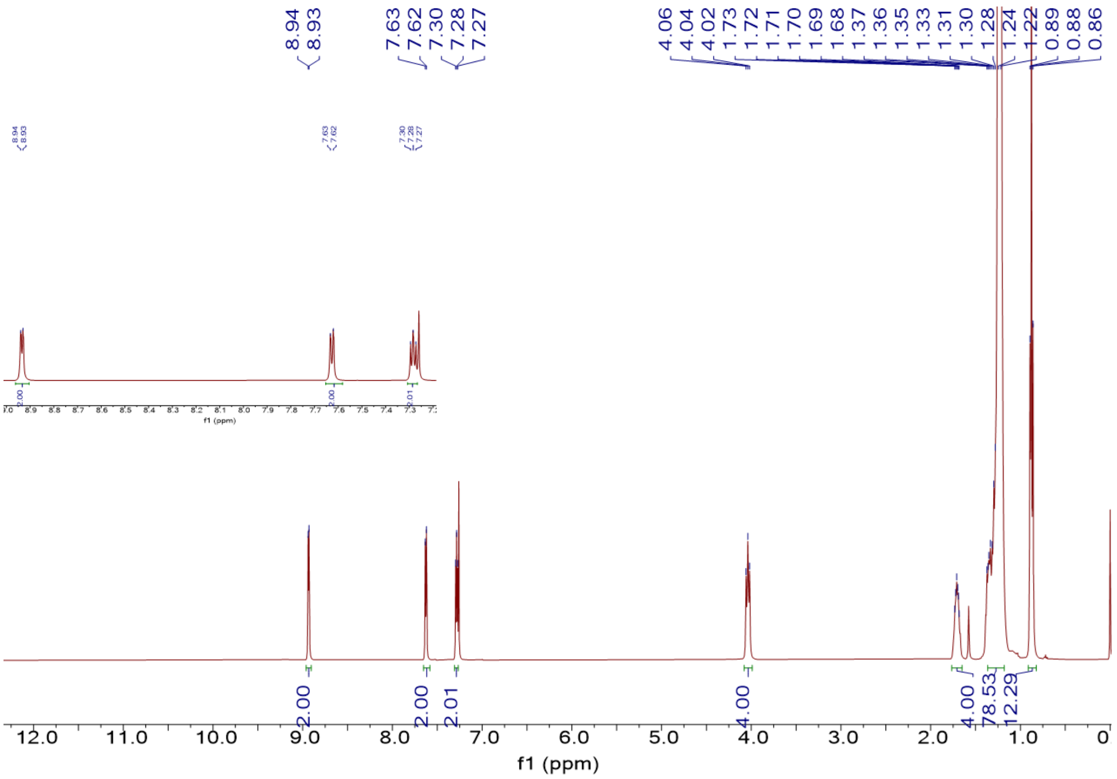

Figure S7. ^{13}C NMR spectra of Compound 2.

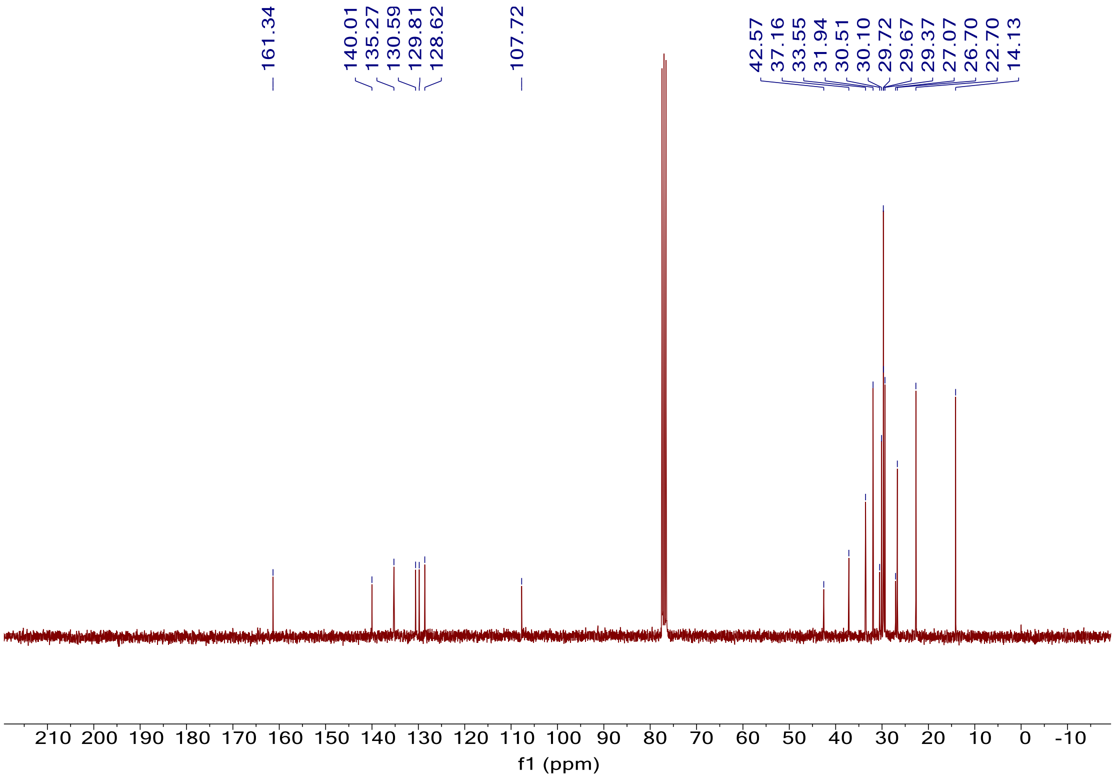

Figure S8. ^{1}H NMR spectra of Compound 3.

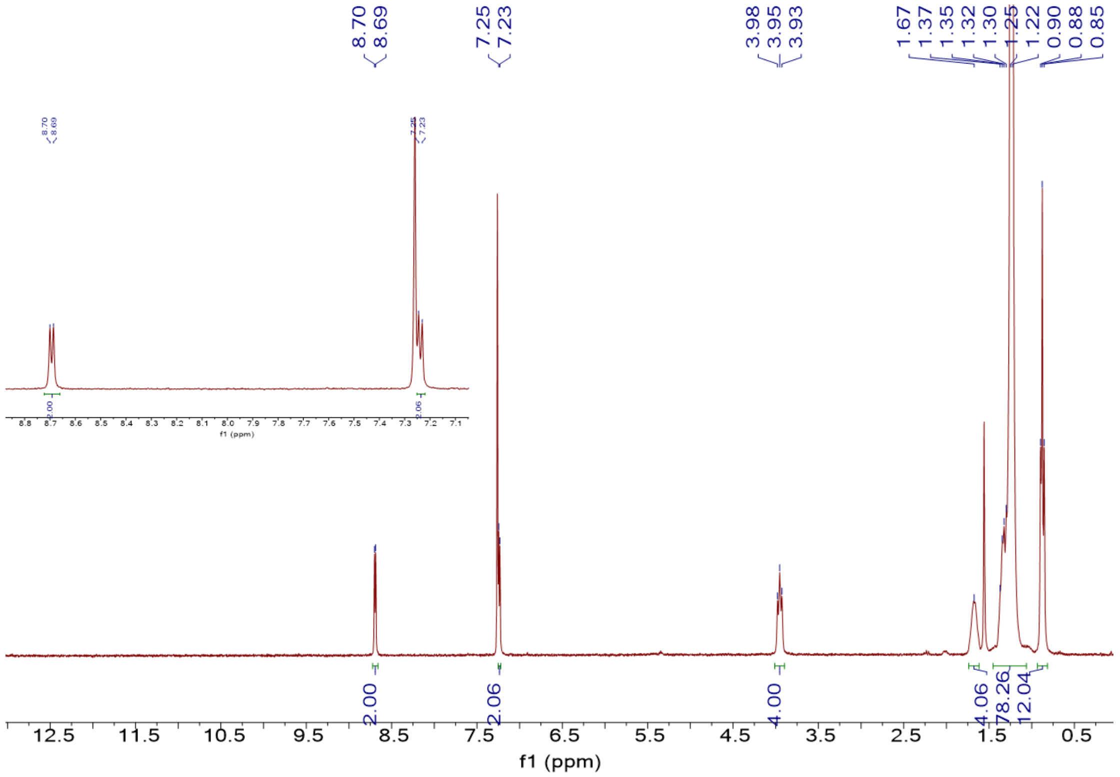

Figure S9. ^{13}C NMR spectra of Compound 3.

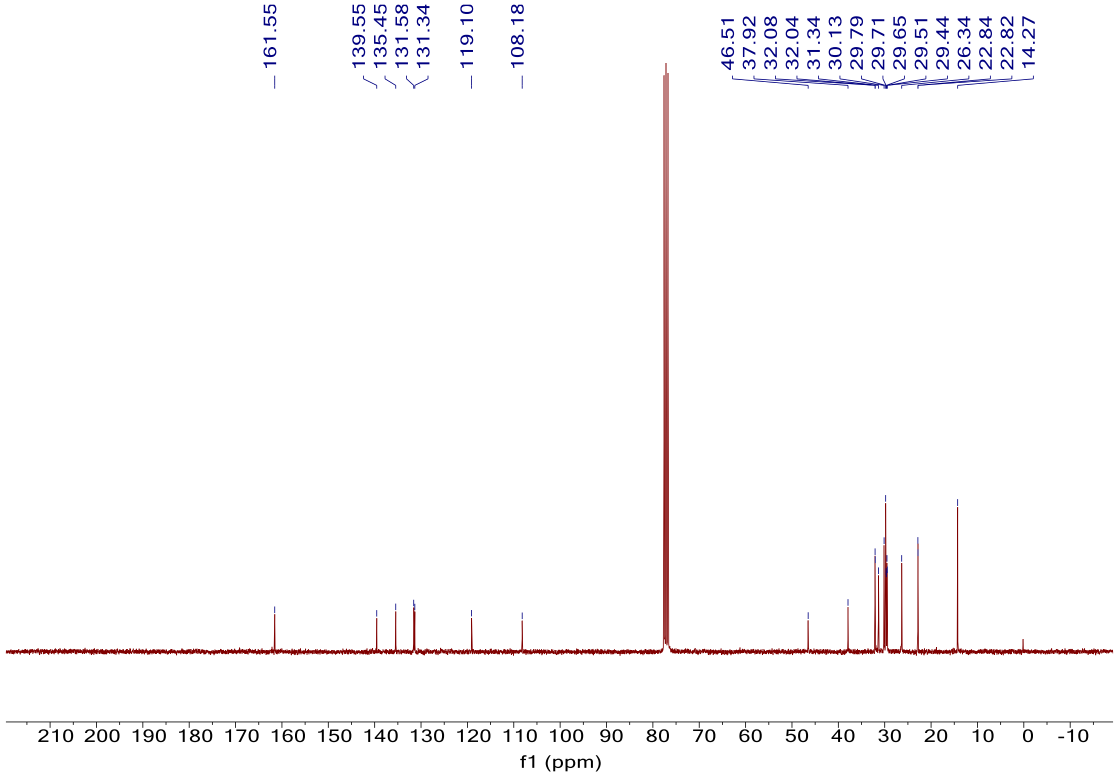

Supplement: Supplementary file 1 [file polymers-15-03392-s001.zip › polymers-2560859-supplementary.pdf]
